# Combination metformin and liraglutide in PCOS: clinical efficacy in women and preclinical insights from gut microbiome modulation in rats

**DOI:** 10.3389/fendo.2025.1599879

**Published:** 2025-11-26

**Authors:** Xue-Feng Long, Yu-Qing Fang, Yan-Hui Li, Jing-Yi Li, Xiu-Ping Wang, Xiao-Li Wang, Ling Zhang, Yi Liu

**Affiliations:** Department of Obstetrics and Gynecology, Union Hospital, Tongji Medical College, Huazhong University of Science and Technology, Wuhan, Hubei, China

**Keywords:** polycystic ovary syndrome, metformin, liraglutide, metabolism, endocrine, gut microbiota

## Abstract

**Background:**

Metformin and liraglutide have been gradually used in the treatment of polycystic ovary syndrome (PCOS) due to their metabolic benefits, but also with some adverse reactions. Evidence suggests that gut microbiota imbalance plays an important role in the pathogenesis of PCOS. This study comprised a clinical trial to evaluate the efficacy of metformin, liraglutide, and their combination in PCOS women, and a parallel animal experiment to explore the potential involvement of gut microbiota.

**Methods:**

In an open-label randomized controlled trial, sixty overweight/obese women with PCOS were randomized to: the MET group received oral metformin (0.85 g twice daily; n=20), the LIRA group received subcutaneous liraglutide (1.2 mg once daily; n=20), and the COM group received both treatments (n=20) for 12 weeks. In a separate animal study, female Sprague-Dawley rats were divided into five groups: (1) PCOS model group (letrozole 1 mg/kg orally); (2) MET group (letrozole + metformin 200 mg/kg orally); (3) LIRA group (letrozole + liraglutide 0.2 mg/kg subcutaneously); (4) COM group (letrozole + metformin + liraglutide at above doses); and (5) healthy controls (no treatment). All treatments lasted 4 weeks.

**Results:**

In the clinical trial, women in MET, LIRA, and COM groups showed significant reductions in body weight, blood glucose, blood lipid, and the LH/FSH ratio. Notably, body weight, BMI, visceral fat area, and body fat percentage decreased more significantly in the COM group than in the MET group (P<0.05). Compared with the MET group, the COM group was more effective in reducing free testosterone (P=0.01). In the animal experiment, the body weight, estrus cycle, and ovarian morphology of rats in the COM group were significantly improved. Letrozole-induced PCOS rats showed intestinal flora disorder, which was improved by metformin, liraglutide, and their combination by altering the alpha and beta diversity and relative abundance of the gut microbiota.

**Conclusion:**

Metformin combined with liraglutide significantly improved metabolic and endocrine characteristics in PCOS women. The associated amelioration of gut microbiota dysbiosis in PCOS rats suggests a potential mechanistic link, which warrants verification in future clinical studies.

## 1 Background

PCOS is a common reproductive endocrine disease that affects 5-18% of women of reproductive age (1). It is characterized by ovulation disorders and hyperandrogenism, accompanied by insulin resistance, type 2 diabetes mellitus, obesity, hyperlipidemia, and infertility (2). More than half of the PCOS women were obese, mainly abdominal obesity (3). Obesity plays an important role in the pathophysiology of PCOS (4). Obesity is manifested as the accumulation of visceral fat, abnormal structure and function of adipose tissue, leading to insulin signaling disorder, increased androgen secretion, and low-grade chronic inflammation, which further aggravate obesity, forming a vicious circle (5, 6). Therefore, weight control is of great value in the treatment of PCOS.

Metformin, as an insulin sensitizer, reduces insulin resistance and hyperinsulinemia, which is commonly used in the treatment of PCOS with insulin resistance (7). Liraglutide is a long-acting glucagon-like peptide-1 (GLP-1) analogue, which decreases food intake by delaying gastric emptying and inhibiting appetite (8). It can significantly reduce body weight in obesity and obesity-related metabolic diseases (9). Our previous study showed that low-dose liraglutide combined with metformin significantly reduced body weight in Chinese Han PCOS patients (10). Currently, the weight loss and metabolic benefits of metformin, low-dose liraglutide, and their combination therapy in overweight PCOS women remain to be further studied.

Gut microbiota, as a huge microbial community in organisms, can generate a variety of metabolites that interact with host cells and regulate a series of physiological metabolic processes, including glucose metabolism, sex hormone metabolism, lipid metabolism, and inflammation (11–14). Metformin is widely used in a variety of metabolic disorders, and gut microbiota is considered to be its potential target (15, 16). Recent studies have shown that liraglutide regulates insulin secretion, reduces body weight, and alleviates inflammation by affecting gut microbiota (17–19). A recent meta-analysis showed that the composition of gut microbiota is altered in PCOS women, supporting the involvement of gut microbiota dysregulation in the endocrine and metabolic disorders of PCOS (20).

In this study, we first demonstrated that the combination of metformin and liraglutide reduced body weight and improved metabolism and endocrine function in PCOS patients through a randomized controlled clinical study. Subsequently, we found that the combination of metformin and liraglutide was effective in the letrozole-induced PCOS model rats. In order to further explore the mechanism of the therapeutic effects of metformin combined with liraglutide in PCOS rats, we then performed 16S rRNA sequencing to detect gut microbiota in rats and found that the combination of metformin and liraglutide significantly improved the composition of gut microbiota, which is expected to be a new therapeutic target for PCOS.

## 2 Materials and methods

### 2.1 Participants

**Figure 1:**
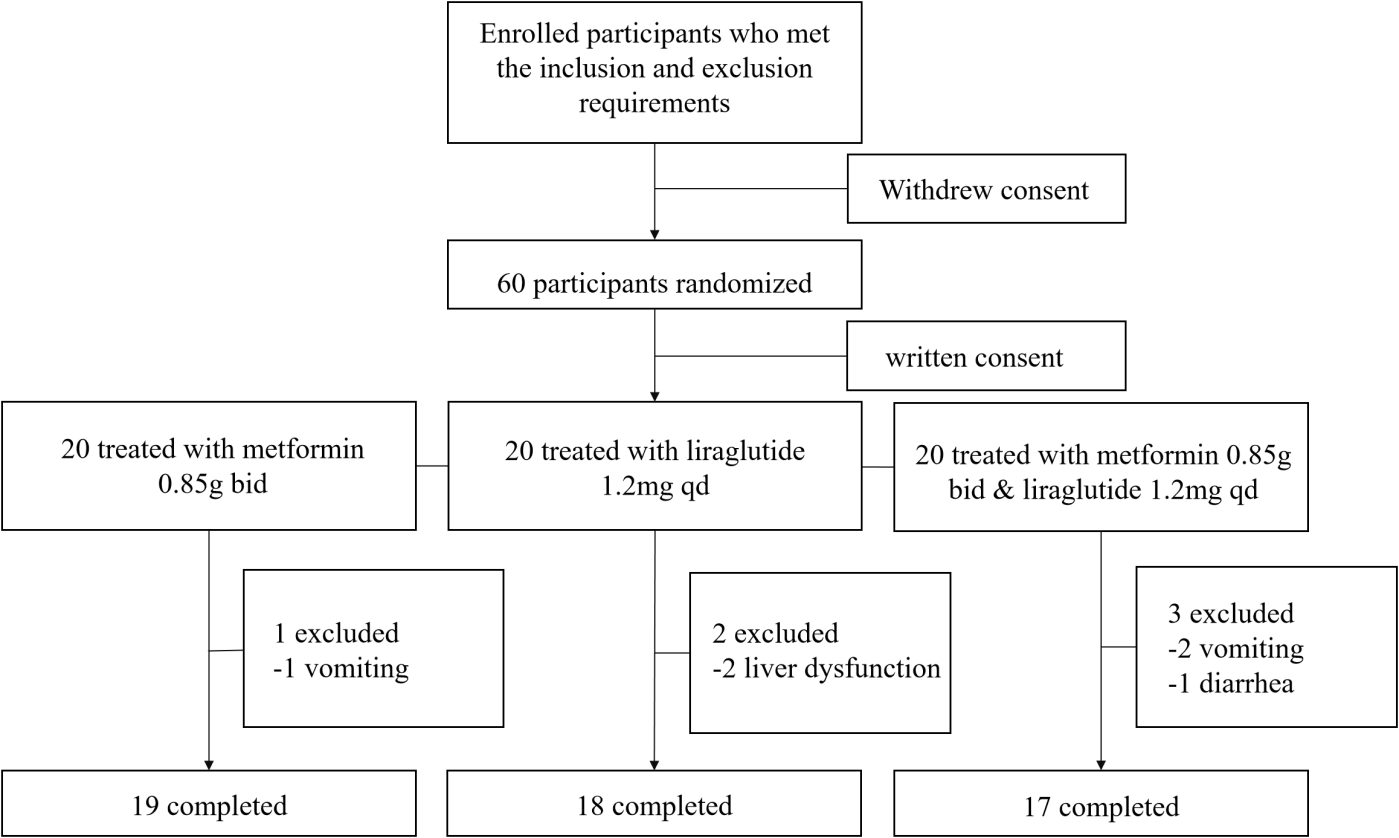
Overview of women included in the study.

Between September 2018 and March 2019, participants with PCOS were recruited from the outpatient Department of Obstetrics and Gynecology, Union Hospital. They all underwent a routine follow-up and were previously diagnosed as PCOS by Rotterdam criteria, including the presence of hyperandrogenemia on biochemical or clinical level, menstrual abnormalities, and polycystic ovarian morphology. They were eligible for enrolment if they were more than 18 years old, premenopausal, overweight or obese (BMI≥24kg/m^2^ as overweight and BMI≥28kg/m^2^ as obese for Chinese), and voluntary. Participants with cardiovascular, kidney, hepatic, pituitary, or thyroid disease and patients who used antiandrogen agents, diabetic drugs, glucocorticoids, or other steroid agents within 90 days before the study began were excluded. Written informed consent was obtained from all participants after a preliminary screening but prior to the final confirmation of eligibility and the initiation of any study-related procedures. All 60 participants who subsequently took part in the study provided written consent. The participant flow is shown in **Figure 1**.

This study was approved by the Medical Ethics Committee of Tongji Medical College of Huazhong University of Science and Technology (2018-S390), and it conformed to the requirements of the Declaration of Helsinki formulated by the World Medical Association. It was registered at the Chinese Clinical Trial Registry (www.chictr.org.cn) with the registration number ChiCTR1800017538.

### 2.2 Experimental protocol

The trial was a 12-week, open-label, randomized controlled study. The random number was generated by IBM SPSS Statistic23 software, and the fixed value was 20180626. According to the order of outpatient visits, they were divided into three arms randomly (1:1:1): participants in MET group were treated with metformin 0.85g twice daily (n=20); participants in LIRA group were treated with liraglutide 1.2 mg once daily via subcutaneous injections (n=20); participants in COM group were treated with liraglutide 1.2 mg once daily via subcutaneous injections combined with metformin 0.85g twice daily (n=20). In the LIRA and COM group, participants used liraglutide at a dose of 0.6mg once a day and increased to 1.2mg once daily after 1 week. The participants were followed monthly for 12 weeks. The primary outcomes were the absolute changes from baseline to week 12 in the following measures (assessed at these two time points): body weight, hormonal parameters, lipid profiles, and measures of glucose metabolism. Participants were monitored throughout the study for safety and tolerability. The study medication was to be discontinued, and the participant managed appropriately, if any of the following predefined conditions occurred: clinically significant liver dysfunction (defined as ALT or AST > 3 times the upper limit of normal), renal impairment (defined as eGFR < 60 mL/min/1.73m²), or symptoms suggestive of acute pancreatitis (21–23). And participants may also withdraw midway through the experiment if they wish. Designers generated the random allocation sequence, and implementers enrolled participants and assigned participants to interventions. The implementers and participants knew the interventions, but the people who collected and analyzed the data were blinded to them.

### 2.3 Measurements

Anthropometric measurements, including height, weight, waist-hip ratio, visceral fat area, and body fat percentage, were conducted using bioelectrical impedance analysis at baseline and at the 12-week follow-up visit. And BMI was calculated as the weight(kg) divided by the square of height(m). Recommended by the Obesity Working Group, the International Life Science Association of China, we considered BMI≥24kg/m2 as overweight and BMI≥28kg/m2 as obese. And blood samples were obtained after an overnight fast at baseline and the end of the study for the determination of glucose, insulin, serum hormone, serum lipid, liver function, and kidney function, followed by a standard 75 g oral glucose tolerance test (OGTT). The free androgen index (FAI) was used to reflect the level of active testosterone. FAI= testosterone (nmol/L) *100/sex hormone binding globulin (nmol/L), and the normal value of FAI was 0.7-6.4 28 29. And insulin resistance was assessed by the homeostasis model assessment of insulin resistance (HOMA-IR) score. It was calculated with the following formula: fasting serum insulin (mU/l) * fasting plasma glucose (mmol/l)/22.5.

### 2.4 Estimation of sample size

G*Power version 3.1.9.2 was used to calculate the sample size. Based on the body weight change data from the study by Jensterle et al., we calculated an effect size (Cohen’s *d*) of 0.585 (24). With a significance level (α) of 0.05 and a power (1-β) of 0.95 for an F-test, a total sample size of 51 was indicated. To accommodate an anticipated 15% dropout rate, we enrolled 60 subjects, with 20 randomly assigned to each group.

### 2.5 PCOS rat model

A total of 30 3-week-old female Sprague-Dawley rats weighting 48-60g were purchased from Vital River Laboratory Animal Technology Co., Ltd. (Beijing, China), and housed in the experimental animal center of Huazhong University of Science and Technology with a controlled environment (21 to 25°C, humidity ranges from 40% to 70%, inverted 12-h daylight cycle, sufficient irradiated sterile feed and autoclaved water).

**Figure 2:**
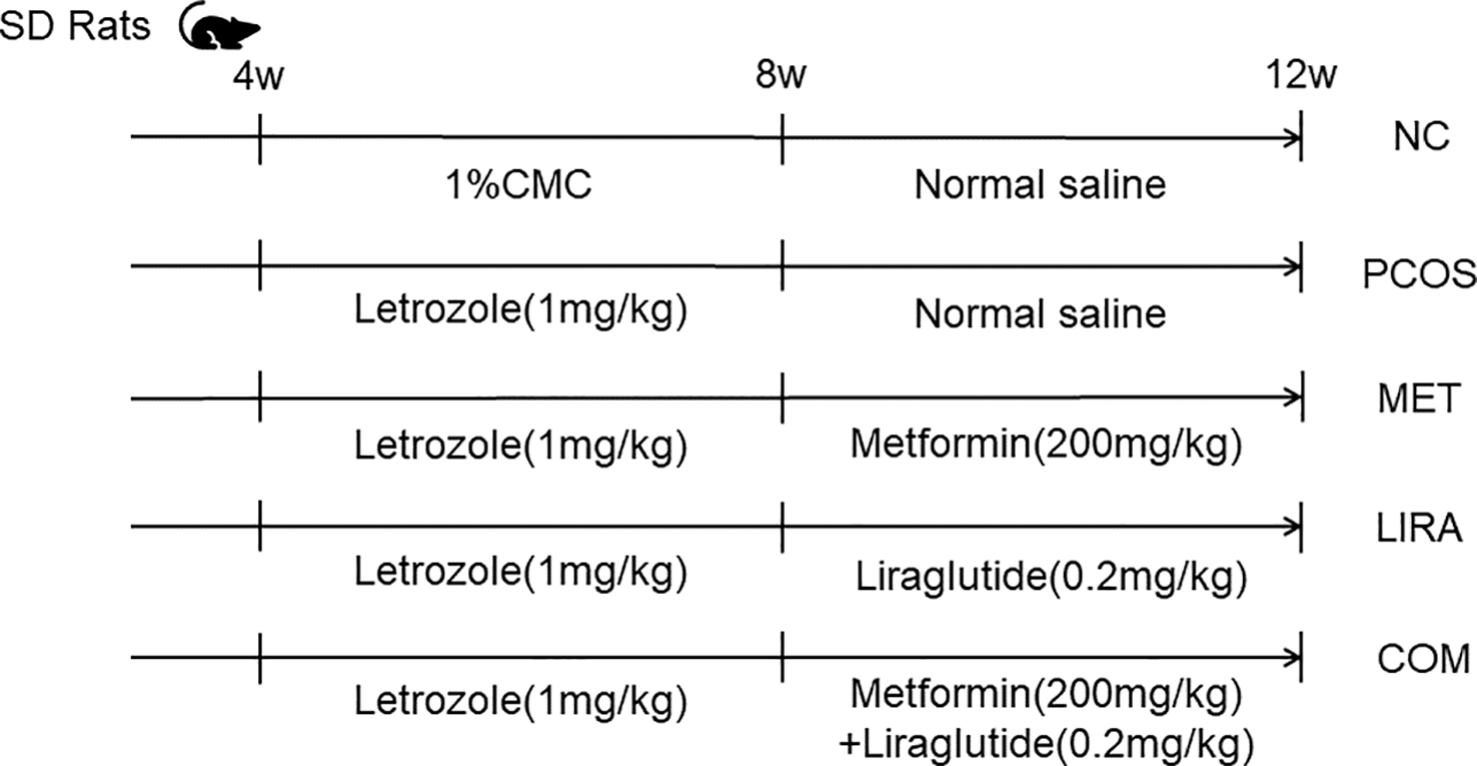
Experimental design and grouping of rats. PCOS, polycystic ovary syndrome; MET, metformin; LIRA, liraglutide; CMC, carboxymethyl cellulose solution.

The experiment began after one week of adaptive feeding. The flow chart of animal experiments is shown in **Figure 2**. Letrozole was dissolved in 1% carboxymethyl cellulose and prepared into a solution with a concentration of 1mg/ml. Rats in the experimental group (n=24) were gavaged with letrozole at a dose of 1mg/kg every day based on the original letrozole rat model study. In contrast, rats in the control group (hereinafter referred to as the NC group, n=6) were gavaged with the same amount of 1% carboxymethyl cellulose at the same time for 4 weeks. The weight of the rats was measured twice a week.

Rats in the experimental group (n=24) were randomly divided into the modeling group (hereinafter referred to as the PCOS group, n=6), the metformin group (hereinafter referred to as the MET group, n=6), the liraglutide group (hereinafter referred to as the LIRA group, n=6) and metformin and liraglutide combined group ((hereinafter referred to as the COM group, n=6) according to their body weight. Metformin was dissolved in saline to prepare a solution of 200mg/mL, and liraglutide was diluted with saline to a solution of 0.2mg/mL. Metformin is given by gavage, while liraglutide is given subcutaneously. The doses of metformin and liraglutide were 200mg/kg and 0.2mg/kg, respectively, administered continuously for 4 weeks. At the end of the experiment, after a night of fasting, feces were collected and transferred to -80°C in time for storage. All animal procedures in this study were approved by the Huazhong University of Science and Technology Animal Care and Use Committee (Protocol Number S14011).

### 2.6 Assessment of serum hormone levels

Rats were anesthetized with isoflurane, and blood was collected from the posterior orbital venous plexus. The collected blood was left at room temperature for two hours, then centrifuged at 3000rpm for 10 minutes, and the supernatant was left to detect the levels of testosterone, estradiol, luteinizing hormone (LH), and follicle-stimulating hormone (FSH) by enzyme-linked immunosorbent assay kits (Ruixin Biotechnology Co., Ltd., Quanzhou, China). The sensitivities of the ELISA kits for testosterone, estradiol, FSH, and LH were 0.1 ng/mL, 0.1 pg/mL, 0.1 IU/L, and 0.1 ng/mL, respectively. The intra- and inter-assay coefficients of variability were <10%.

### 2.7 Oral glucose tolerance test

In the oral glucose tolerance test (OGTT), female rats were fasted for 16h during which time they had free access to water. Blood glucose levels were measured using an Accu-Chek glucose monitor (Roche Diagnostics Corp, Shanghai, China) at 30, 60, 90, and 120 minutes before and after intraperitoneal injection of D-glucose.

### 2.8 Estrous cycle analysis

After two weeks of intervention, vaginal smears from each rat were taken and smeared daily between 12:00 and 13:00 for 10 consecutive days. Slides were observed under a light microscope to assess the stage of the estrous cycle. A high concentration of leukocytes was present in diestrus, and nucleated epithelial cells were predominant in proestrus. while cornified epithelial cells were predominant in estrus. Metestrus was characterized by the presence of both cornified epithelial cells and leukocytes.

### 2.9 Hematoxylin and eosin staining

Ovarian tissues were fixed with 4% paraformaldehyde, embedded in paraffin, and sectioned. The slides were subsequently deparaffinized in xylene and gradient alcohol, followed by being stained with hematoxylin and eosin. The sections were dehydrated with gradient alcohol, sealed with neutral resin, and examined under a microscope.

### 2.10 DNA isolation and 16S rRNA gene sequencing

Bacterial DNA was extracted using the E.Z.N.A.^®^ Soil DNA Kit. And the V3-V4 region of the bacterial 16S rRNA gene was amplified by PCR using the following primer pair: 338F, ACTCCTACGGGAGGCAGCAG, and 806R, GGACTACHVGGGTWTCTAAT. Library construction was performed with the NEXTFLEX Rapid DNA-Seq Kit, and sequencing was performed using Illumina’s MiSeq PE300 platform. The raw sequences were quality-controlled using Trimmomatic software, and the data were optimized by splicing the DNA sequences using FLASH software.

### 2.11 Microbial analysis

OTU clustering was performed on all sequences, and the one with the highest abundance was selected as the representative sequence. The RDP Classifier software was used to annotate the species classification of each sequence, and the alignment threshold was set at 70%. The sequence was compared with the Silva database (SSU128) to classify the species of each OUT sequence. The number of sequences classified at the taxonomic level of each species in each sample was counted and plotted into a bar chart of species composition for the analysis of relative species abundance. Linear discriminant analysis (LDA) was used to estimate the impact of the relative abundance of different species on the differences between the two groups, and LDA ≥ 2.0 was used as a significant difference criterion to identify the communities or species that made significant differences between the two groups (25). Spearman correlation heat maps were drawn using the R language to discover important patterns and relationships among dominant species.

### 2.12 Statistical analysis

In animal experiments, all data were analyzed by IBM SPSS Statistics 23.0 and were presented as mean ± standard error of mean (SEM). For the data consistent with normal distribution, one-way ANOVA was used and followed by *post hoc* comparisons with the Bonferroni test, and the Kruskal-Wallis test was used to compare the data that are not normally distributed. P <0.05 indicates statistical significance. Comparisons between groups were conducted using independent samples t tests or one-way analysis of variance (ANOVA) with Tukey’s *post hoc* test (for continuous variables) or the chi-square test (for categorical variables).

In the clinical study, IBM SPSS Statistics (version 23.0) was used for statistical analyses. Data were analyzed based on the intention-to-treat (ITT) principle, which included all 60 randomly assigned participants in their originally allocated groups. For the 6 participants who discontinued the study, missing outcome data at the end of the trial (week 12) were imputed using the Last Observation Carried Forward (LOCF) method. Normal data distribution was analyzed by the Shapiro–Wilk test. Normally distributed data was presented as mean (standard deviation), and nonnormally distributed data was presented as median (interquartile range). And the one-way ANOVA or Welch’s test with the *post-hoc* Bonferroni’s test or Games-Howell test were used for a precise comparison of the differences amongst the arms. The paired t-test and nonparametric Wilcoxon signed-rank test were used to compare pretreatment and posttreatment values in each of the treatment groups. And the comparison of proportions was evaluated by the Chi-square test. P values of <0.05 were considered statistically significant.

## 3 Results

### 3.1 Baseline characteristics of the participants

There were 54 participants (19 on MET, 18 on LIRA, 17 on COM) who completed the research and were included in the study. Baseline characteristics of the study population are provided in **Table 1**. Because of randomization, there were no significant differences at baseline in any of the parameters among the treatment groups.

**Table 1:** Baseline of indexes in each group.

Index	Metformin	Liraglutide	Combination	P
Weight (kg)	69.20 (8.32)	67.51 (7.69)	69.24 (7.72)	0.758
BMI (kg/m2)	28.10 (2.95)	27.94 (2.59)	26.68 (2.14)	0.219
Waist-to-hip ratio	0.89 (0.06)	0.90 (0.03)	0.91 (0.04)	0.601
Visceral fat area (cm2)	138.12 (35.73)	131.47 (31.27)	129.71 (26.69)	0.699
Percentage of body fat (%)	40.30 (4.73)	39.56 (4.44)	38.12 (3.93)	0.332
FSH (IU/L)	6.06 (1.38)	6.87 (2.46)	6.29 (1.43)	0.391
LH (IU/L)	10.45 (6.12)	12.26 (6.57)	12.79 (5.55)	0.481
PRL (ng/ml)	18.00 (8.20)	17.35 (6.09)	18.70 (9.39)	0.883
TT (nmol/L)	1.36 (0.91)	1.56 (0.61)	1.99 (0.72)	0.05
FT (pmol/L)	8.63 (3.28)	9.65 (2.81)	11.71 (4.88)	0.052
SHBG (nmol/L) ^*^	28.29 (22.17, 74.42)	34.33 (29.99, 50.15)	31.46 (25.08, 59.50)	0.923
LH/FSH	1.72 (0.84)	1.88 (0.98)	2.09 (0.94)	0.482
FAI	4.76 (4.48)	4.51 (2.53)	5.85 (3.59)	0.517
TC (mmol/L)	4.30 (0.67)	4.52 (0.76)	4.92 (1.02)	0.083
HDL-C(mmol/L)	1.22 (0.19)	1.26 (0.33)	1.28 (0.33)	0.793
LDL-C(mmol/L)	2.71 (0.59)	2.73 0.68)	3.04 (0.81)	0.283
TG (mmol/L)	1.36 (0.69)	1.85 (1.26)	1.74 (1.01)	0.302
LDL-C/HDL-C	2.27 (0.56)	2.30 (0.81)	2.54 (1.03)	0.57
TC/HDL-C	3.59 (0.66)	3.82 (1.11)	4.05 (1.36)	0.443
Glu 0 min OGTT (mmol/L)	5.65 (0.66)	5.63 (0.76)	5.80 (0.73)	0.739
Glu 30 min OGTT (mmol/L)	10.24 (1.85)	9.83 (1.95)	10.01 (1.22)	0.774
Glu 60 min OGTT (mmol/L)	10.85 (3.40)	10.66 (3.51)	10.25 (2.30)	0.848
Glu 120 min OGTT (mmol/L)	8.94 (2.83)	8.33 (2.34)	7.74 (1.57)	0.311
Glu 180 min OGTT (mmol/L) ^*^	5.20 (3.90, 6.50)	5.85 (4.70, 7.73)	4.20 (3.70, 6.75)	0.097
AUC Glucose (min* mmol/L)	26.31 (6.67)	25.78 (6.05)	24.46 (4.07)	0.618
Insulin 0 min OGTT (μIU/L)	16.53 (7.50)	22.02 (10.97)	17.94 (8.75)	0.181
Insulin 30 min OGTT (μIU/L) ^#^	109.06 (57.08)	188.50 (151.21)	147.05 (67.25)	0.149
Insulin 60 min OGTT (μIU/L) ^*^	122.01 (83.16, 202.52)	143.72 (81.76, 268.93)	137.41 (102.77, 193.71)	0.782
Insulin 120 min OGTT (μIU/L) ^*^	106.49 (52.93, 241.31)	9.64 (56.71, 262.26)	121.20 (53.34, 156.86)	0.909
Insulin 180 min OGTT (μIU/L) ^*^	37.17 (20.72, 74.39)	63.89 (26.70, 148.81)	27.69 (12.34, 59.78)	0.082
AUC Insulin (min*μIU/L) ^*^	285.07 (180.34, 477.97)	355.81 (200.29, 684.04)	331.84 (237.69, 412.36)	0.626
HbA1c (%)	5.35 (0.38)	5.28 (0.40)	5.26 (0.34)	0.773
HOMA-IR ^*^	4.38 (2.42, 5.19)	4.75 (3.57, 6.67)	4.12 (2.98, 6.31)	0.396

Results are presented as mean (standard deviation, SD) or median (25th percentile-75th percentile, p25–p75).

^#^ indicates that the data does not meet the homogeneity of variance, and Welch’s test is adopted.

^*^ indicates that the data does not conform to the normal distribution, and Kruskal-Wallis test is adopted.

### 3.2 Metabolic changes before and after treatment among the treated populations

**Table 2** presents the values of metabolic parameters at baseline and week 12 for all treatment groups, along with the results of within-group comparisons. Inter-group comparisons of the absolute changes in metabolic parameters from baseline to week 12 are provided in **Table 3**. All treatment teams experienced reductions in body weight at the first post-baseline visit at Week 12. Participants in the COM group lost on average 7.42(3.24) kg compared with a 5.19(2.28) kg loss in participants of the LIRA group and a 3.87(2.88) kg loss in the MET group (P = 0.02 for the differences between the COM and MET groups). In terms of weight loss percentage, the average weight loss was 5.28% in the MET group, 7.71% in the LIRA group, and 10.55% in the COM group. Clinically significant (≥5%) weight reduction was achieved in 10 of the participants in the MET group (52.63%), 14 of the participants in the LIRA group (77.78%), and 16 of the participants in the COM group (94.12%), with statistically significant differences among the three groups (P = 0.025). The results of pairwise comparisons suggested that the percentage of those who achieved significant weight loss was higher in the COM group than in the MET group(P<0.05). Moreover, BMI decreased by 1.84(1.88) kg/m2 in the MET group compared with 2.18(0.99) kg/m2 in the LIRA group and 2.91(1.21) kg/m2 in the COM group. The waist-to-hip ratio and visceral fat area were significantly decreased in participants from the LIRA and COM groups. The changes in waist-to-hip ratio and visceral fat ratio were statistically significant among the three groups, with P < 0.01 for the differences between the COM and MET groups.

**Table 2:** Comparison of metabolic and hormonal parameters before and after treatment within each group.

Index	Metformin			Liraglutide			Combination		
	Before	After	P	Before	After	P	Before	After	P
Weight (kg)	69.20 (8.32)	65.33 (6.28)	**p<0.001**	67.51 (7.69)	62.32 (7.57)	**p<0.001**	69.24 (7.72)	61.82 (6.36)	**p<0.001**
BMI (kg/m2)	28.10 (2.95)	26.25 (2.23)	**0.001**	27.94 (2.59)	25.76 (2.52)	**p<0.001**	26.68 (2.14)	23.77 (1.91)	**p<0.001**
Waist-to-hip ratio	0.89 (0.06)	0.90 (0.04)	0.115	0.90 (0.03)	0.88 (0.03)	**0.039**	0.91 (0.04)	0.87 (0.04)	**p<0.001**
Visceral fat area (cm2)	138.12 (35.73)	130.16 (32.11)	**0.027**	131.47 (31.27)	111.93 (29.59)	**p<0.001**	129.71 (26.69)	102.59 (23.45)	**p<0.001**
Percentage of body fat (%)	40.30 (4.73)	38.99 (4.89)	0.340	39.56 (4.44)	36.98 (4.45)	**0.001**	38.12 (3.93)	35.13 (3.66)	**p<0.001**
FSH (IU/L)	6.06 (1.38)	6.67 (1.85)	0.272	6.87 (2.46)	5.98 (2.35)	0.194	6.29 (1.43)	7.03 (2.05)	0.154
LH (IU/L)	10.45 (6.12)	8.24 (3.10)	0.199	12.26 (6.57)	7.94 (4.13)	**0.025**	12.79 (5.55)	9.05 (2.71)	**0.03**
PRL (ng/ml) *^#^*	18.00 (8.20)	22.15 (13.18)	0.215	17.35 (6.09)	24.62 (17.76)	**0.045**	18.70 (9.39)	19.35 (8.22)	0.723
T (nmol/L)	1.36 (0.91)	1.42 (0.71)	0.638	1.56 (0.61)	1.12 (0.53)	**0.002**	1.99 (0.72)	1.40 (0.30)	**0.004**
FT (pmol/L)	8.63 (3.28)	6.96 (2.53)	**0.021**	9.65 (2.81)	5.88 (2.67)	**0.001**	11.71 (4.88)	6.36 (2.20)	**p<0.001**
SHBG (nmol/L)	28.29 (22.17, 74.42)	167.20 (56.80, 200.00)	**0.002**	34.33 (29.99, 50.15)	153.20 (119.45, 200.00)	**p<0.001**	31.46 (25.08, 59.50)	155.90 (88.74, 184.50)	**0.001**
LH/FSH	1.72 (0.84)	1.30 (0.52)	0.057	1.88 (0.98)	1.29 (0.47)	**0.033**	2.09 (0.94)	1.40 (0.66)	**0.018**
FAI	4.76 (4.48)	1.89 (1.79)	**0.005**	4.51 (2.53)	0.87 (0.64)	**p<0.001**	5.85 (3.59)	1.15 (0.58)	**p<0.001**
TC (mmol/L)	4.30 (0.67)	4.72 (0.66)	**0.001**	4.52 (0.76)	4.46 (0.68)	0.599	4.92 (1.02)	4.89 (0.84)	0.911
HDL-C(mmol/L) ^#^	1.22 (0.19)	1.44 (0.26)	**0.027**	1.26 (0.33)	1.39 (0.19)	**0.049**	1.28 (0.33)	1.53 (0.36)	**p<0.001**
LDL-C(mmol/L)	2.71 (0.59)	2.77 (0.56)	**p<0.001**	2.73 0.68)	2.63 (0.55)	0.444	3.04 (0.81)	2.86 (0.70)	0.359
TG (mmol/L)	1.36 (0.69)	1.63 (0.59)	0.656	1.85 (1.26)	1.43 (0.86)	**0.031**	1.74 (1.01)	1.74 (1.09)	0.976
LDL-C/HDL-C	2.27 (0.56)	1.97 (0.50)	0.102	2.30 (0.81)	1.92 (0.43)	**0.014**	2.54 (1.03)	1.96 (0.58)	**0.006**
TC/HDL-C ^#^	3.59 (0.66)	3.35 (0.55)	0.081	3.82 (1.11)	3.26 (0.57)	**0.005**	4.05 (1.36)	3.34 (0.80)	**0.005**
Glu 0 min OGTT (mmol/L)	5.65 (0.66)	5.51 (0.65)	0.373	5.63 (0.76)	5.53 (0.83)	0.715	5.80 (0.73)	5.32 (0.44)	**0.038**
Glu 30 min OGTT (mmol/L) ^#^	10.24 (1.85)	9.70 (1.36)	0.139	9.83 (1.95)	9.17 (1.92)	0.242	10.01 (1.22)	9.32 (1.89)	0.128
Glu 60 min OGTT (mmol/L)	10.85 (3.40)	10.17 (2.72)	0.087	10.66 (3.51)	9.26 (2.94)	0.079	10.25 (2.30)	9.40 (2.85)	0.102
Glu 120 min OGTT (mmol/L)	8.94 (2.83)	8.40 (2.64)	**p<0.001**	8.33 (2.34)	7.16 (2.08)	**0.045**	7.74 (1.57)	7.39 (2.35)	0.509
Glu 180 min OGTT (mmol/L)	5.20 (3.90, 6.50)	5.10 (3.90, 6.70)	0.632	5.85 (4.70, 7.73)	4.90 (4.28, 5.93)	**0.02**	4.20 (3.70, 6.75)	4.40 (3.85, 5.35)	0.218
AUC Glucose (min* mmol/L)	26.31 (6.67)	24.90 (4.92)	0.085	25.78 (6.05)	22.63 (4.87)	**0.028**	24.46 (4.07)	25.20 (11.22)	0.746
Insulin 0 min OGTT (μIU/L)	16.53 (7.50)	16.90 (6.69)	0.795	22.02 (10.97)	17.31 (8.29)	**0.001**	17.94 (8.75)	14.80 (6.29)	0.126
Insulin 30 min OGTT (μIU/L)	109.06 (57.08)	97.56 (52.18)	0.268	188.50 (151.21)	139.56 (99.37)	0.099	147.05 (67.25)	117.71 (57.04)	0.09
Insulin 60 min OGTT (μIU/L) ^#^	122.01 (83.16, 202.52)	95.25 (52.76, 175.76)	0.053	143.72 (81.76, 268.93)	152.94 (69.25, 215.81)	0.396	137.41 (102.77, 193.71)	136.96 (78.66, 200.79)	0.381
Insulin 120 min OGTT (μIU/L) ^#^	106.49 (52.93, 241.31)	86.41 (63.70, 147.66)	**p<0.001**	9.64 (56.71, 262.26)	84.79 (44.88, 163.40)	0.071	121.20 (53.34, 156.86)	90.2 (57.07,184.35)	0.687
Insulin 180 min OGTT (μIU/L) ^#^	37.17 (20.72, 74.39)	42.29 (16.83, 52.14)	0.629	63.89 (26.70, 148.81)	44.76 (10.97, 57.38)	**0.002**	27.69 (12.34, 59.78)	16.04 (9.11, 42.58)	0.062
AUC Insulin (min*μIU/L) ^#^	285.07 (180.34, 477.97)	232.97 (162.07, 390.33)	0.099	355.81 (200.29, 684.04)	273.08 (171.67, 495.41)	0.022	331.84 (237.69, 412.36)	327.12 (192.80, 404.58)	0.356
HbA1c (%) ^#^	5.35 (0.38)	5.20 (0.38)	0.081	5.28 (0.40)	4.88 (0.39)	**p<0.001**	5.26 (0.34)	4.96 (0.29)	**p<0.001**
HOMA-IR	4.38 (2.42, 5.19)	3.59 (2.67,5.95)	0.936	4.75 (3.57, 6.67)	4.26 (2.62, 5.50)	**0.02**	4.12 (2.98, 6.31)	3.75 (2.35,4.79)	0.093

Results are presented as mean (standard deviation, SD) or median (25th percentile-75th percentile, p25–p75).

^#^ indicates that the data were nonnormally distributed, and nonparametric Wilcoxon signed-rank test is adopted. P values <0.05 were marked bold, indicating statistical significance.

**Table 3:** Comparison of absolute changes in metabolic and hormonal parameters before and after treatment among treatment groups.

Index	Metformin	Liraglutide	Combination	P
Weight (kg)	-3.87 (2.96)	-5.19 (2.34)	-7.42 (3.34)	**P=0.002, M VS C = 0.002**
BMI (kg/m^2^)	-1.84 (1.93)	-2.18 (1.02)	-2.91 (1.24)	0.096
Waist-to-hip ratio	0.01 (0.03)	-0.01 (0.03)	-0.03 (0.03)	**P<0.001, M VS L = 0.032, M VS C<0.001**
Visceral fat area (cm^2^)	-7.96 (14.46)	-19.53 (15.94)	-27.11 (16.74)	**P=0.002, M VS C = 0.002**
Percentage of body fat (%)	-1.31 (2.47)	-2.58 (2.58)	-2.99 (2.53)	0.119
FSH (IU/L)	0.19 (2.21)	-0.65 (3.70)	0.74 (3.47)	0.432
LH (IU/L)	-3.41 (7.96)	-3.36 (5.77)	-3.40 (7.19)	0.999
PRL (ng/ml) ^*^	3.25 (-1.83, 6.32)	2.44 (-1.23, 9.28)	0.52 (-3.98, 3.81)	0.762
TT (nmol/L)	-0.04 (0.61)	-0.29 (0.52)	-0.42 (0.50)	0.12
FT (pmol/L)	-1.12 (3.85)	-2.51 (3.52)	-4.81 (3.28)	**P=0.012, M VS C = 0.01**
SHBG (nmol/L)	72.56 (76.18)	92.18 (63.40)	89.20 (44.77)	0.6
LH/FSH	-0.49 (1.03)	-0.47 (0.81)	-0.84 (1.42)	0.558
FAI	-2.84 (3.48)	-3.83 (3.96)	-2.89 (2.16)	0.605
TC (mmol/L) ^#^	0.24 (0.97)	0.20 (0.44)	-0.11 (0.57)	0.193
HDL-C(mmol/L)	0.20 (0.19)	0.25 (0.18)	0.15 (0.30)	0.473
LDL-C(mmol/L)	-0.04 (0.75)	0.00 (0.51)	-0.30 (0.49)	0.288
TG (mmol/L)	0.15 (0.96)	0.15 (1.02)	-0.21 (0.78)	0.426
LDL-C/HDL-C	-0.39 (0.71)	-0.38 (0.49)	-0.52 (0.54)	0.747
TC/HDL-C	-0.41 (0.87)	-0.50 (0.56)	-0.49 (0.70)	0.825
Glu 0 min OGTT (mmol/L)	-0.28 (0.61)	0.23 (0.94)	-0.47 (0.78)	**P=0.029, L VS C = 0.032**
Glu 30 min OGTT (mmol/L)	-0.23 (1.59)	-0.62 (2.31)	-1.22 (1.36)	0.267
Glu 60 min OGTT (mmol/L)	-0.38 (1.76)	-1.04 (2.74)	-0.90 (2.70)	0.689
Glu 120 min OGTT (mmol/L)	-0.24 (2.09)	-0.58 (1.78)	-1.38 (2.49)	0.271
Glu 180 min OGTT (mmol/L)	-0.18 (1.81)	-0.43 (1.66)	-1.38 (1.70)	0.105
AUC Glucose (min* mmol/L)	-0.81 (3.58)	-1.83 (4.51)	-3.48 (5.03)	0.197
Insulin 0 min OGTT (μIU/L)	-1.04 (5.70)	-1.74 (7.00)	-4.96 (6.09)	0.154
Insulin 30 min OGTT (μIU/L)	-11.80 (42.78)	-36.20 (93.25)	-22.21 (86.21)	0.628
Insulin 60 min OGTT (μIU/L)	-30.29 (67.21)	-37.57 (129.64)	-1.73 (81.38)	0.514
Insulin 120 min OGTT (μIU/L)	-30.89 (87.39)	-25.12 (94.16)	-37.47 (83.53)	0.919
Insulin 180 min OGTT (μIU/L) ^*^	0.26 (-19.80, 6.55)	-26.15 (-86.35, -14.92)	-5.92 (-29.59, 3.98)	0.139
AUC Insulin (min*μIU/L) ^*^	-9.97 (-193.31, 32.34)	-59.54 (-174.52, 4.57)	-1.97 (-147.67, 37.86)	0.969
HbA1c (%)	-0.11 (0.33)	-0.30 (0.31)	-0.36 (0.25)	**P=0.03, M VS C = 0.036**
HOMA-IR	-0.43 (1.41)	-0.32 (2.21)	-1.64 (1.95)	0.082

Results are presented as mean (standard deviation, SD) or median (25th percentile-75th percentile, p25–p75).

^#^ indicates that the data does not meet the homogeneity of variance, and Welch’s test is adopted.

^*^ indicates that the data does not conform to the normal distribution, and Kruskal-Wallis test is adopted. P values <0.05 were marked bold, indicating statistical significance.

In addition, the level of HDL-C was improved(P<0.05) in all groups, while LDL-C/HDL-C and TC/HDL-C ratios decreased (P<0.05) in LIRA and COM groups. As for blood glucose metabolism, a significant reduction in fasting blood glucose levels (P<0.05) was observed in the COM group, and the LIRA group demonstrated significant reductions in fasting insulin (P<0.05) and in postprandial plasma glucose at both 120 and 180 minutes (P<0.05). In the MET group, a significant reduction was specifically observed in postprandial plasma glucose at 120 min (P < 0.05). As for the HbA1c level, it decreased significantly in both the LIRA and COM groups, with a greater reduction observed in participants in the COM group (P = 0.03) compared with those in the MET group (P = 0.036). According to previous studies, we defined insulin resistance as HOMA-IR ≥2.69 (26). At the time of enrollment, there were 14, 16, and 14 participants with insulin resistance in the MET, LIRA, and COM groups, respectively. After 12 weeks, the number of participants with insulin resistance was 14, 14, and 9, as shown in **Table 4**. The effective rate of insulin resistance in the LIRA group was 12.5%, while it was 35.71% in the COM group. There were statistical differences among the three groups (P = 0.033), particularly between COM and MET.

**Table 4:** Comparison of effectiveness for insulin resistance in each group.

Group	Total number	Ineffective	Effective	Chi square test	
				chi2	p
Metformin	14	14 (100%)	0 (0)	6.225	0.033
Liraglutide	16	14 (87.5%)	2 (12.5%)		
Combination	14	9 (64.29%)	5 (35.71%) ^*^		

^*^ means a statistical difference compared with MET.

### 3.3 Endocrine changes before and after treatment among the treated populations

As shown in **Tables 2**, **3**, significant reductions in plasma levels of free testosterone, as well as significant increases in plasma levels of SHBG, were observed in all participants at the end of the research. Additionally, LH and testosterone levels decreased in the LIRA and COM groups (P < 0.05). The significant difference between the three treatment groups was the level of free testosterone (P = 0.012), with a reduction that was significantly greater in participants in the COM group compared with those in the MET group (P = 0.01). The number of people whose LH/FSH ≥2 in the MET group decreased from 6 to 2, while in the LIRA group, it decreased from 10 to 2, and in the COM group, it decreased from 8 to 2. The number of participants with hyperandrogenemia (FAI ≥6.4) in the LIRA and COM groups decreased from 5 to 0, while the number in the MET group decreased from 5 to 1. According to the Chi-square test, compared with the baseline, the proportions of people with FAI >6.4 and LH/FSH >2 were both significantly decreased; however, there was no significant difference among the three groups.

### 3.4 Adverse events among the treated populations

The most frequent adverse events in participants were mild or moderate gastrointestinal reactions. Adverse events associated with MET were nausea (6/19), vomiting (4/19), diarrhea (4/19), dyspepsia (1/19), anorexia (6/19), and insomnia (1/19). Participants in the LIRA group had nausea (9/18), vomiting (3/18), diarrhea (4/18), anorexia (1/18), headache (1/18), hypoglycemia (1/18), liver dysfunction (1/18), and rash at the injection site (1/18). And in the COM group, participants had nausea (9/17), vomiting (5/17), diarrhea (6/17), dyspepsia (2/17), anorexia (2/17), constipation (1/17), headache (1/17), hypoglycemia (1/17), and liver dysfunction (1/17). The majority of these adverse events were mild and tolerable, subsiding gradually within two weeks.

### 3.5 The effects of metformin, liraglutide, and their combination on metabolic and endocrine disorders in PCOS rats

**Figure 3:**
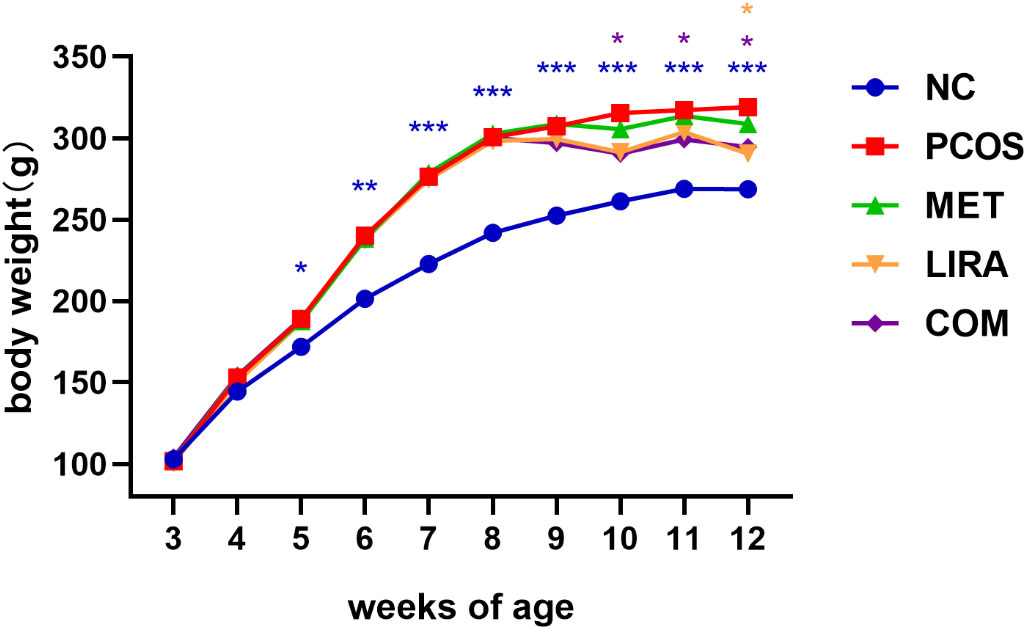
Changes in the mean body weight of rats in each group from 3 weeks of age were compared with the PCOS group in each group. *P < 0.05, **P < 0.01, ***P < 0.001. PCOS, polycystic ovary syndrome; MET, metformin; LIRA, liraglutide.

**Figure 4:**
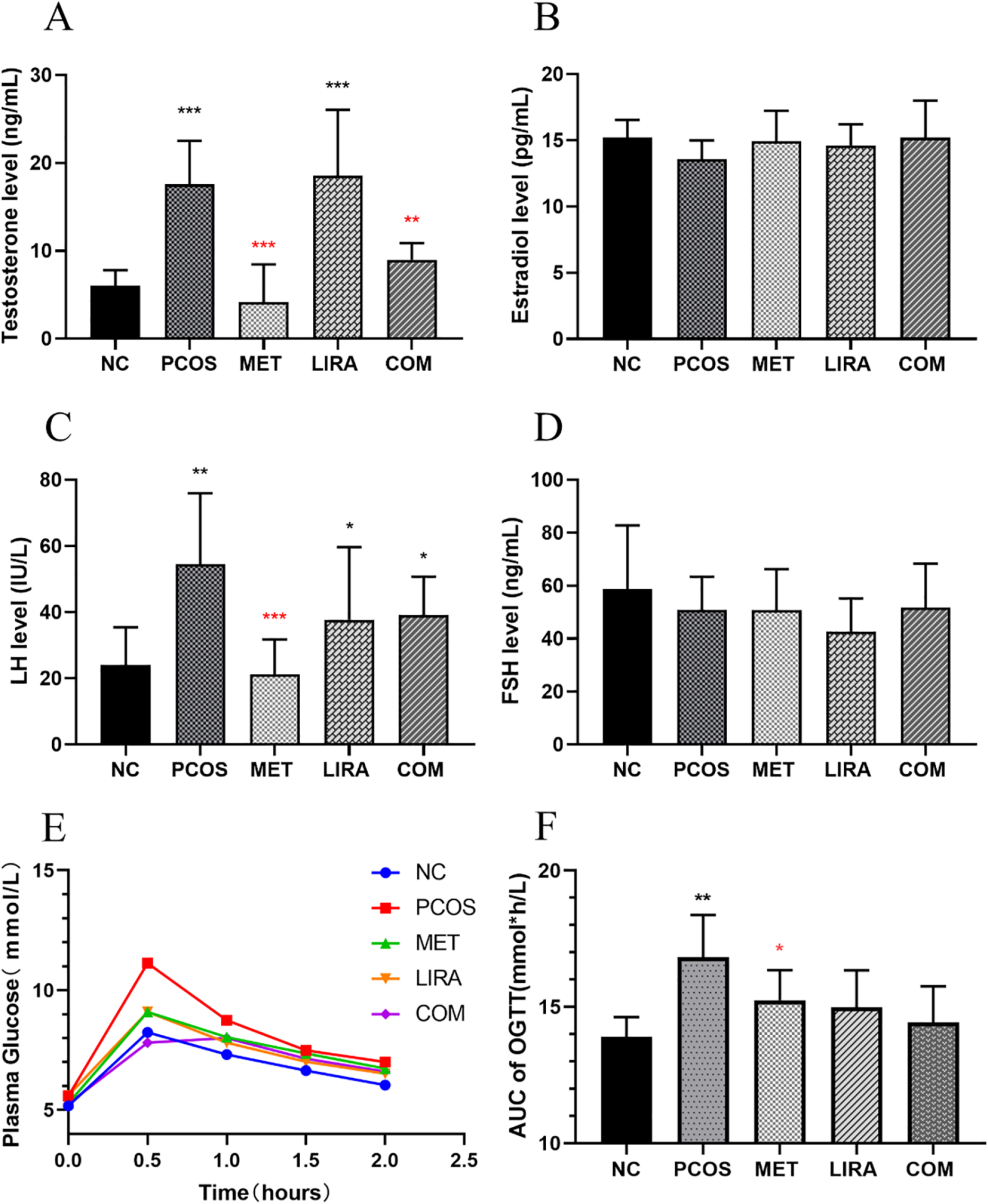
Comparisons of serum sex hormones and blood glucose at 12 weeks. The concentrations of testosterone **(A)**, estradiol **(B)**, LH **(C)** and FSH **(D)** in the serum were quantified with ELISA kit. **(E)** Changes in the mean plasma glucose during OGTT in each group; **(F)** The mean of AUC of plasma glucose during OGTT in each group. The data are expressed as means ± SEM. * indicates a significant difference compared with NC group, * indicates significant difference compared with PCOS group. *P < 0.05, **P < 0.01, ***P < 0.001. PCOS, polycystic ovary syndrome; MET, metformin; LIRA, liraglutide.

The flow chart of animal experiments is shown in **Figure 2**. From the 5th week to the 8th week, the body weight of rats treated with letrozole by gavage was significantly higher than that of controls. Subsequent administration of a combination of MET and LIRA for two weeks resulted in a marked weight loss that persisted until 12 weeks of age. Rats in the LIRA group also showed significant weight loss at 12 weeks of age. However, there was no significant difference in body weight between the MET group and the PCOS group until the end of the treatment (**Figure 3**). The elevated levels of testosterone and LH are essential endocrine characteristics of PCOS (27). The serum testosterone and LH levels in the PCOS group were significantly higher than those in the NC group. We found that the levels of testosterone were down-regulated in the MET and COM groups. In contrast, the level of LH decreased only in the MET group, which suggested that metformin and its combination with liraglutide have a specific corrective effect on the hormone disorder of PCOS rats. Still, liraglutide had no significant effect (**Figures 4A–D**). PCOS is often accompanied by insulin resistance, which is manifested as impaired glucose tolerance (28). OGTT experiments showed that the blood glucose of PCOS rats was higher than that of the control group at each time point, while MET, LIRA, and their combination reduced the blood glucose level and improved glucose tolerance (**Figures 4E, F**).

### 3.6 Metformin, liraglutide, and their combination reversed estrous cycle disorders and ovarian performance in PCOS rats

**Figure 5:**
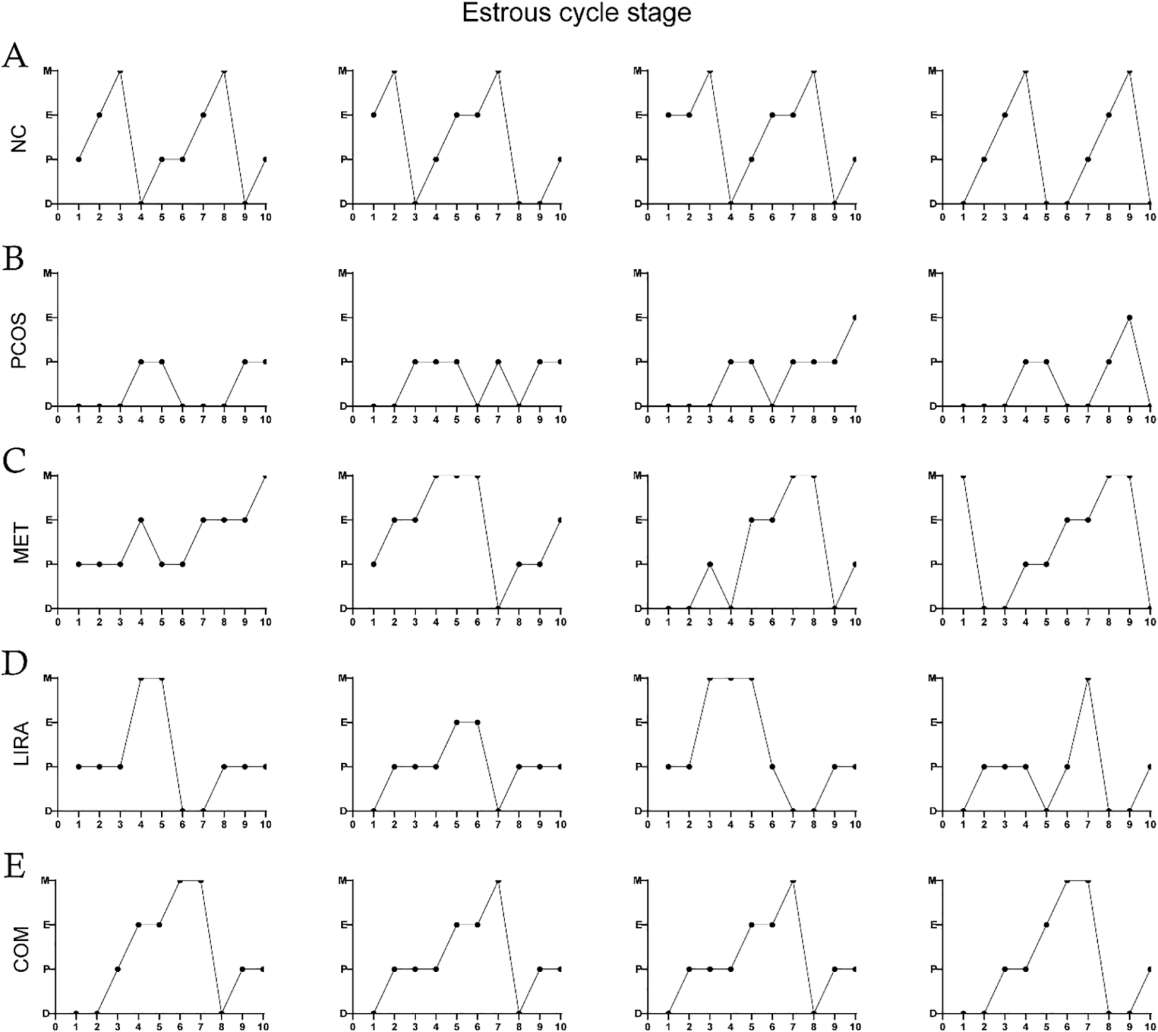
Estrous cycles in each group. Estrous cycles in the NC group **(A)**, PCOS group **(B)**, MET group **(C)**, LIRA group **(D)**, COM group **(E)**. PCOS, polycystic ovary syndrome; MET, metformin; LIRA, liraglutide.

**Figure 6:**
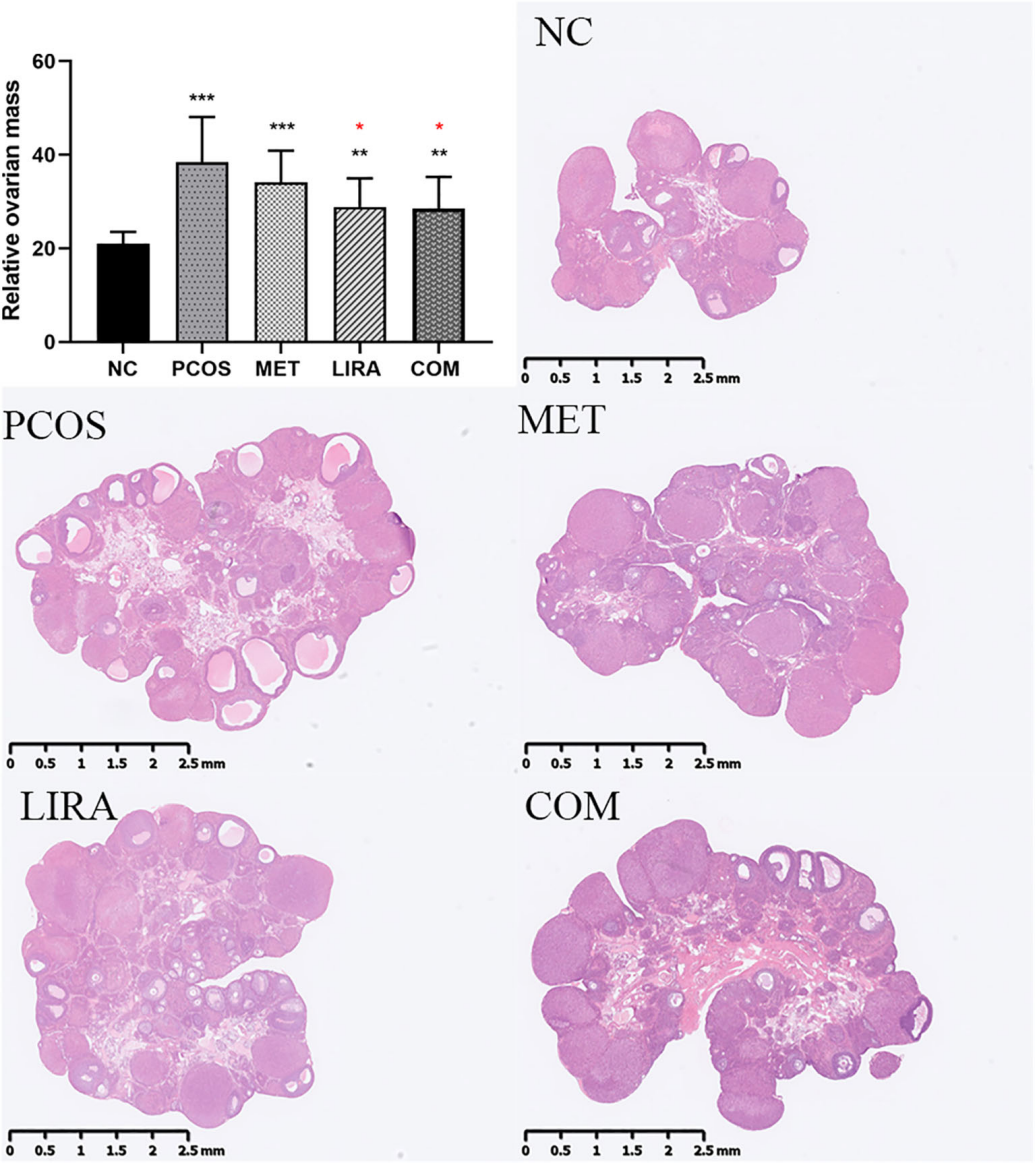
The mean relative ovarian mass of each group and the maximum cross-sectional view of the ovaries of rats in each group under HE staining. The data are expressed as means ± SEM. * indicates a significant difference compared with the NC group, * indicates a significant difference compared with the PCOS group. *P < 0.05, **P < 0.01, ***P < 0.001. PCOS, polycystic ovary syndrome; MET, metformin; LIRA, liraglutide.

Oligo-ovulation is a key feature of PCOS, which can be reflected in the disturbance of the estrous cycle in PCOS rat models (29, 30). As shown in **Figures 5A–E**, the estrous cycle of rats in the MET group, LIRA group, and COM group was recovered compared with that in the PCOS group. Apparently, metformin combined with liraglutide almost reversed the irregular estrous cycle in PCOS rats, which was close to the normal level. Bilateral ovarian mass/body weight *10^5^ was used to obtain the relative ovarian mass. According to **Figure 6**, the relative ovarian mass of the PCOS group was significantly higher than that of the control group. In contrast, the treatment of liraglutide and liraglutide combined with metformin decreased ovarian mass in PCOS rats. Polycystic ovarian changes are an important pathological feature of PCOS (2). The morphology of the ovary showed that there was increased ovarian volume, cystic follicles, and decreased granulosa cell layers of the follicle wall in the PCOS group. While ovarian morphology in the MET group, LIRA group, and COM group was recovered, as reflected by reduced ovarian volume, alleviated polycystic changes, and increased corpus luteum, suggesting the recovery of ovarian function.

### 3.7 Metformin, liraglutide, and their combination changed the diversity of gut microbiota in PCOS rats

**Figure 7:**
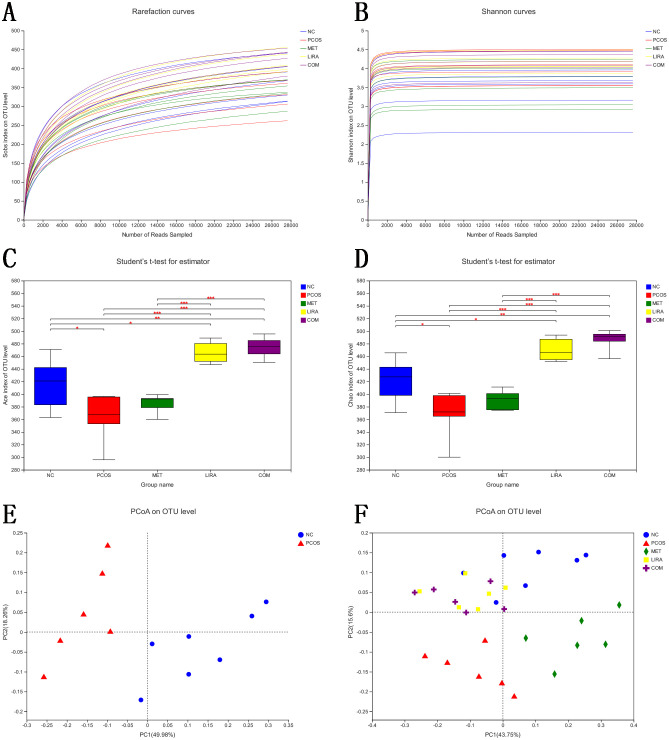
Comparisons of alpha-diversity and beta-diversity among different groups. **(A)** Sobs curves of all samples. **(B)** Shannon-Wiener curves of all samples. **(C)** statistical plots of ACE index of all groups. **(D)** statistical plots of Chao index of all groups. **(E)** PCoA analysis based on weighted_unifracde algorithm for NC and PCOS group colonies. **(F)** PCoA analysis based on weighted_unifracde algorithm for all groups. PCOS, polycystic ovary syndrome; MET, metformin; LIRA, liraglutide; PC, principle coordinate.

To determine the effects of liraglutide and metformin on the structure of gut microbiota in PCOS rats, we performed the principal coordinates analysis (PCOA) at the OUT level on the five groups based on the weighted_unifrac algorithm (**Figures 7A–F**). The composition distances of gut microbiota between the NC and PCOS groups were dispersed, while the composition distances of samples in the same group were relatively concentrated, suggesting that the structure of the bacterial community was different between the two groups. The ANOSIM similarity analysis demonstrated that there was a significant difference in β-diversity between NC and PCOS groups (F = 6.727, R2 = 0.379, P = 0.003). According to **Figure 7F**, MET had a great change in PC1 principal component, while the change degree of the LIRA group and the COM group were relatively similar, and the two groups had an obvious change in PC2 principal component. Moreover, the beta diversity of the LIRA and COM groups was relatively close to that of the NC group. The ACE index and Chao index, commonly used to represent the richness of species community, were significantly decreased in the PCOS group compared with the NC group (P<0.05), indicating a lower alpha diversity in letrozole-treated female rats compared with placebo rats. And the alpha diversity of LIRA and COM groups was significantly higher than that of the PCOS group (P<0.05).

### 3.8 Metformin and liraglutide recovered the imbalanced gut microbiota community in PCOS rats

**Figure 8:**
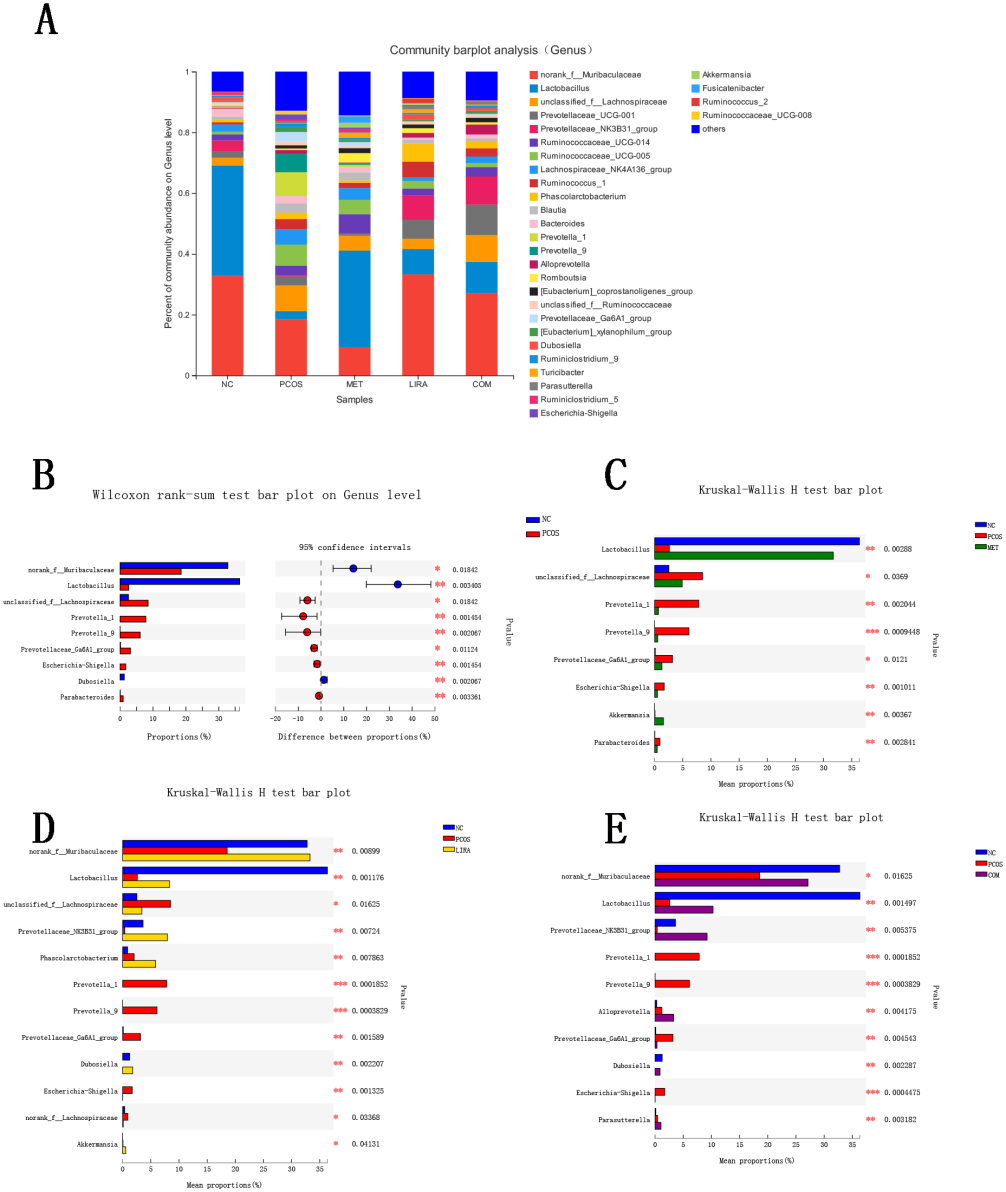
Community barplot analysis and comparison of the compositional differences at genus level among different groups. **(A)** Histogram of differences in flora composition between groups. **(B)** Wilcoxon rank sum test comparing the differences in intestinal flora between NC and PCOS groups. **(C)** Kruskal-Wallis rank sum test comparing the differences in flora composition between NC, PCOS, and MET groups. **(D)** Kruskal-Wallis rank sum test comparing the differences in flora composition between NC, PCOS, and LIRA groups. **(E)** Kruskal-Wallis rank sum test comparing the differences in flora composition between NC, PCOS, and COM groups. *P < 0.05, **P < 0.01. PCOS, polycystic ovary syndrome; MET, metformin; LIRA, liraglutide.

We subsequently assessed species and relative abundance of bacteria at the genus level in the different groups (**Figure 8**). As shown in **Figure 8A**, the top ten florae were Muribaculaceae, Lactobacillus, Lachnosiraceae, Prevotellaceae_UCG-001, Prevotellaceae_NK3B31_group, Prevotellaceae_UCG-014, Prevotellaceae_UCG-005, Lachnospiraceae_NK4A136_group, Ruminococcus_1, and Phascolarctobacterium. Compared to the NC group, the abundances of norank_f_Muribaculaceae, Lactobacillus, and Dubosiella were significantly lower (P<0.05) in the PCOS group by the Wilcoxon rank sum test. At the same time, unclassified_f_Lachnospiraceae, Prevotella_1, Prevotella_9, Prevotellaceae_Ga6A1_group, Escherichia-Shigella, and Parabacteroides genera were significantly increased (P<0.05). Compared with the PCOS group, there was an increased abundance of Lactobacillus (P<0.05) but a decreased abundance of Prevotella_1, Prevotella_9, Prevotellaceae_Ga6A1_group, Escherichia-Shigella in the MET, LIRA, and COM groups (P<0.05). In addition, the relative abundance of Akkermansia significantly increased in the MET group, Phascolarctobacterium and Akkermansia significantly increased in the LIRA group, and Alloprevotella and Parasutterella significantly increased in the COM group (P<0.05). The relative abundance of unclassified_f_Lachnospiraceae and Parabacteroides in the MET group significantly decreased, and the relative abundance of unclassified_f_Lachnospiraceae and norank_f_Lachnospiraceae in the LIRA group also decreased significantly (P < 0.05).

### 3.9 The correlation between the intestinal microbes and metabolism parameters of PCOS

**Figure 9:**
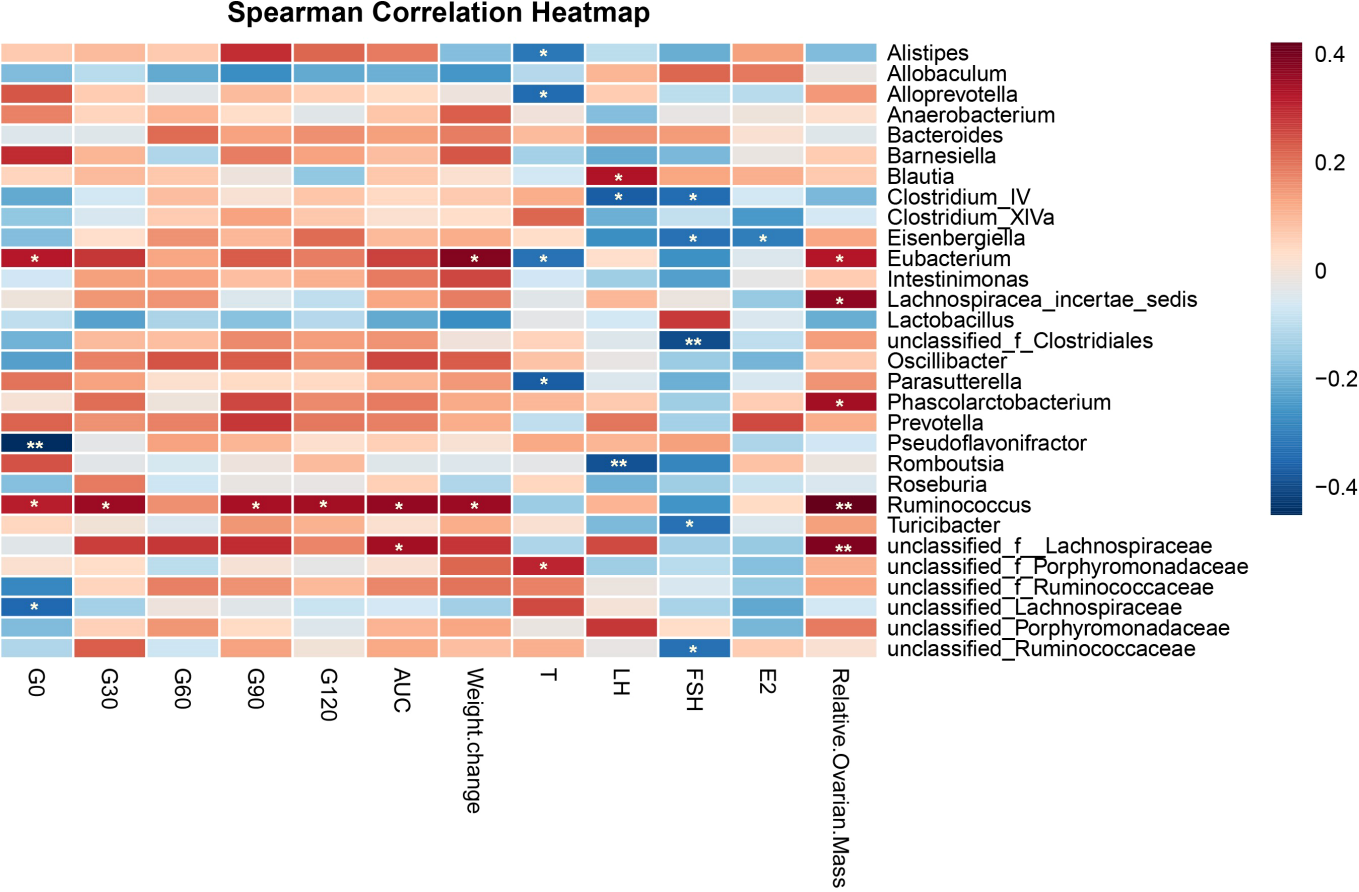
Spearman correlation heatmap showing the association of genus-level abundance data with metabolism parameters of Letrozole-induced rat model. Metabolic parameters are on the X-axis and species are on the Y-axis. The x-axis and y-axis are metabolism parameters and species, respectively. As in the legend on the right, different colors represent different R values. *P < 0.05, **P < 0.01.

To explore whether specific microbes were responsible for the metabolism parameters, we performed correlation analysis at the genus level (**Figure 9**). Ruminococcus was significantly positively correlated with blood glucose, weight, and relative ovarian mass. Unclassified_f_Lachnospiraceae was negatively associated with AUC, which was increased in the PCOS group. Unclassified_f_Porphyromonadaceae was significantly positively correlated with testosterone, while Alistipes, Alloprevotella, Eubacterium, and Parasutterella were negatively related to testosterone. Alloprevotella and Parasutterella were decreased in the PCOS group and were elevated in the COM group, which indicated that the combination of metformin and liraglutide might reduce testosterone by affecting the relative abundance of Alloprevotella and Parasutterella.

## 4 Discussion

PCOS is a complicated and highly heterogeneous endocrine disease that greatly affects women’s reproductive health, metabolism, and psychology (31). Obesity is closely related to the occurrence and development of PCOS (32). Women with PCOS are more likely to be overweight or obese, and obesity in turn can aggravate hyperinsulinemia and hyperandrogenemia, key clinical features of PCOS (33–35). Clinical studies have shown that weight loss can significantly improve the reproductive, hyperandrogenic, and metabolic characteristics of PCOS (36). According to international evidence-based guidelines, weight management is currently the primary treatment strategy for PCOS (37).

Weight management includes improving lifestyle and pharmacological treatment. Pharmacological treatment is more effective than lifestyle modification and requires less patient compliance (38). Metformin is widely used in the treatment of insulin resistance in PCOS due to its ability to improve glucose metabolism, but the effect on weight loss is limited (39). Liraglutide can significantly reduce body weight and improve insulin sensitivity, showing great prospects in the treatment of PCOS, but more studies are still needed to verify this (40, 41). Studies have shown that liraglutide combined with metformin can reduce body weight and improve gonadal and metabolic profiles in overweight patients with PCOS (10, 42). Consistent with these findings, we found that the combination of metformin and liraglutide significantly reduced body weight and reduced SHBG and FAI in overweight PCOS patients compared with metformin and liraglutide alone. However, our study found that liraglutide combined with metformin reduced the LH/FSH ratio, while He et al. and Long et al. found that the combination of liraglutide and metformin only decreased LH, and there was no significant difference in the LH/FSH ratio (42, 43). We speculated that this might be related to the different doses of metformin and liraglutide used and the differences in study populations. Notably, we found that baseline androgen levels varied among the treatment groups, with total and free testosterone approaching statistical significance (P = 0.05 and P = 0.052, respectively; **Table 1**). Interestingly, the COM group exhibited the highest numerical values for both TT and FT at baseline. Rather than confounding the results, this baseline profile indicates a robust therapeutic capacity of the combination therapy to overcome a potentially greater hyperandrogenic burden. Interestingly, the MET group exhibited a distinct baseline profile with concurrently low SHBG and free testosterone, which reflected the heterogeneity of PCOS. The fact that metformin significantly reduced free testosterone and increased SHBG levels even from this unique baseline profile strongly demonstrates its potent therapeutic effect.

Furthermore, Wang et al. observed that liraglutide combined with metformin reduced body weight, improved estrous cycle, reversed ovarian polycystic morphology, and corrected sex hormone disorders in PCOS rats, which is supported by our study (44). A number of studies supported that metformin and liraglutide alone could improve PCOS by changing gut microbiota and metabolites (45–47). Studies have shown that alpha diversity of gut microbiota was significantly decreased in PCOS patients (48, 49). Similarly, we found decreased alpha diversity of gut microbiota in PCOS model rats, which is consistent with Wang et al (48). Decreased α diversity affects intestinal permeability and leads to weight gain, which is closely associated with metabolic abnormalities in PCOS (50, 51). In addition, reduced alpha diversity was widely found in inflammation-related diseases, suggesting that it is associated with the chronic low-grade inflammatory state (52, 53). Beta diversity describes the compositional differences by qualitative and quantitative assessments in different samples. Compared with the healthy control group, PCOS patients have a significant change in the β diversity of gut microbiota, but some studies have not found this difference (54, 55). The inconsistent findings across studies may be related to study protocols, the sample size, use of antibiotics, nutrition, or some other factors (56). In our study, both PCoA principal component analysis and ANOSIM analysis showed a major separation in beta diversity between the PCOS and NC groups.

As for the genus level of the intestinal flora, we found the relative abundance of beneficial bacteria, such as Muribaculaceae, Dubosiella, and Lactobacillus, significantly decreased, while the relative abundance of harmful bacteria, including Prevotella and Escherichia-Shigella, was significantly higher in PCOS rats compared with rats in the NC group. Changes in the abundance of Muribaculaceae, Lactobacillus, Prevotella, and Escherichia-Shigella have a positive effect on the weight gain of PCOS. Muribaculaceae, as butyrate-producing bacteria, play a vital role in maintaining the barrier function of the internal mucus layer, which is associated with antioxidant effects in colitis mice (57, 58). It has been shown that the abundance of Muribaculaceae was decreased in the gut of mice fed with a high-fat diet (HFD), which was negatively correlated with body weight, fat mass, and glucose metabolism (59). The significant weight loss of rats in the MET, LIRA, and COM groups at week 12 may be related to the increased abundance of Lactobacillus and the decreased abundance of Prevotella and Escherichia in the gut. Lactobacillus is an important probiotic in the gut microbiome, which can directly kill pathogens by producing lactic acid, acetic acid, propionic acid, bacteriocin, and reactive oxygen species (ROS), balance the integrity of the intestinal barrier, and improve the host immune response (60, 61). Moreover, Yue et al. observed that Lactobacillus enhanced microbial richness and diversity, simultaneously changing the diversity of the intestinal microbiota, thus playing an anti-obesity effect in HFD mice (62). Escherichia-Shigella is a proinflammatory opportunistic pathogen that was enriched in the gut of diabetic and obese patients (63–65). Some studies have observed that Escherichia-Shigella significantly increased in the gut of PCOS women (66, 67). Decreased insulin sensitivity is an important pathological feature of PCOS. Gut microbiota can affect insulin sensitivity by affecting the level of branched short-chain amino acids (BCAA). Elevated BCAA levels were associated with insulin resistance and type 2 diabetes (68, 69). Bacteria from the genus Prevotella produced BCAA; therefore, an increase in the genus Prevotella can lead to insulin resistance (70). Furthermore, the body weight loss induced by Met and LIRA treatment in rats was also related to the increased abundance of Akkermansia. Akkermansia is an intestinal mucus-enhancing bacterium involved in maintaining the intestinal barrier and regulating glucose metabolism (71, 72). The relative abundance of Akkermansia was negatively correlated with the development of obesity and type 2 diabetes (73). In addition, we found that the abundance of Phascolarctobacterium was increased in the gut of rats in the LIRA group. In previous studies, Phascolarctobacterium, a SCFA-producing bacterium, has been positively associated with the metabolic status of the host (74). The decline of Phascolarctobacterium is correlated with enhanced intestinal permeability, inflammation, and nutritional imbalance (75). Thus, the improvement of metabolism by LIRA was related to its increase in the abundance of Phascolarctobacterium. Interestingly, we found that the abundance of Alloprevotella and Parasutterella increased after the treatment of MET combined with LIRA. Alloprevotella, as a SCFA-producing bacterium, has anti-inflammatory effects and is negatively correlated with metabolic syndrome (76, 77). Parasutterella is a beneficial bacterium involved in the regulation of abnormal inflammation and metabolism (78). Chronic low-grade inflammation plays an important role in the pathogenesis of polycystic ovary syndrome, contributing to hormonal abnormalities and metabolic disorders (79). Therefore, MET and LIRA increased the abundance of Alloprevotella and Parasutterella, which might promote its improvement in metabolism and endocrinology in PCOS rats.

There are some limitations that need to be elucidated in the study. Firstly, the analysis of gut microbiota was conducted exclusively in an animal model. Therefore, although our clinical trial confirms the efficacy of the combination therapy of metformin and liraglutide in PCOS women, we cannot directly attribute this benefit to gut microbiota modulation. In our animal experiment, the combination of metformin and liraglutide induced significant changes in the gut microbiota of PCOS rats. This leads us to hypothesize that the clinical benefits observed with this therapy may be mediated through the amelioration of gut microbiota. Moreover, the specific dysbiosis pattern observed in women with PCOS, which was characterized by decreased Lactobacillus and increased Escherichia-Shigella, was significantly improved in the COM group of our rat model, providing direct support for our hypothesis (80–82). Nevertheless, it is necessary to directly analyze the gut microbiota of PCOS patients receiving combination therapy with metformin and liraglutide. Future studies should aim to correlate specific bacterial taxa with clinical outcomes in human cohorts. Secondly, the clinical trial was conducted with an open-label design, which may introduce performance and detection bias, as the lack of blinding for participants and clinicians could influence subjective endpoints such as treatment adherence and data recording. However, the primary outcomes, including body weight, BMI, visceral fat area, hormonal and metabolic parameters, are objective and were analyzed by blinded personnel, thus mitigating this risk. Nonetheless, future studies employing a double-blind, placebo-controlled design would be valuable to confirm our findings. Thirdly, the sample size (n=6 per group) is relatively small for the gut microbiota analysis, which is characterized by high interindividual variability. This limits the power to detect significant differences, particularly among low-abundance microbes. Fourthly, the lifestyle management required in the study population could not be fully standardized, which may affect the final results. Finally, the causal relationship between gut microbiota and metabolism and reproduction needs to be verified by further experiments, such as a fecal microbiota transplantation experiment, to clarify the role of gut microbiota in the pathogenesis of PCOS.

## 5 Conclusions

In summary, our study demonstrated that the combined administration of MET and LIRA improved the metabolic and reproductive endocrine characteristics of PCOS patients, which provided some implications for guiding the clinical treatment of PCOS. Meanwhile, co-administration of metformin and liraglutide corrected metabolic and endocrine abnormalities and improved ovarian morphology in LE-induced PCOS rats. Microbial sequencing results of rat feces showed that metformin, liraglutide, and their combination had beneficial effects on reversing the disorder of intestinal flora in LE-induced PCOS rats, supporting the important role of intestinal flora in the pathogenesis and treatment of PCOS. Taken together, our findings provide valuable insights into the pathological mechanism of PCOS and also demonstrate the efficacy of the combination therapy of metformin and liraglutide in the treatment of PCOS.

## Data Availability

The original contributions presented in the study are included in the article/supplementary material. Further inquiries can be directed to the corresponding author/s.
